# Spatiotemporal association of low birth weight with Cs-137 deposition at the prefecture level in Japan after the Fukushima nuclear power plant accidents: an analytical-ecologic epidemiological study

**DOI:** 10.1186/s12940-020-00630-w

**Published:** 2020-07-09

**Authors:** Hagen Scherb, Keiji Hayashi

**Affiliations:** 1 Helmholtz Zentrum München, German Research Center for Environmental Health, Institute of Computational Biology, Ingolstädter Landstr. 1, D-85764 Neuherberg, Germany; 2 Hayashi Children’s Clinic, 4-6-11-1F Nagata, Joto-ku Osaka-Shi, Osaka, 536-0022 Japan

**Keywords:** Gestational development, Live births, Nuclear accidents, Pregnancy outcome, Radionuclide deposition, Dose-rate, Radiation induced genetic effects

## Abstract

**Background:**

Perinatal mortality increased in contaminated prefectures after the Fukushima Daichi Nuclear Power Plant (FDNPP) accidents in Japan in 2011. Elevated counts of surgeries for cryptorchidism and congenital heart malformations were observed throughout Japan from 2012 onward. The thyroid cancer detection rate (2011 to 2016) was associated with the dose-rate at the municipality level in the Fukushima prefecture. Since the birth weight is a simple and objective indicator for gestational development and pregnancy outcome, the question arises whether the annual birth weight distribution was distorted in a dose-rate-dependent manner across Japan after Fukushima.

**Methods:**

The Japanese Ministry of Health, Labour, and Welfare provides prefecture-specific annual counts for 26.158 million live births from 1995 to 2018, of which 2.366 million births (9.04%) with weights < 2500 g. Prefecture-specific spatiotemporal trends of the low birth weight proportions were analyzed. Logistic regression allowing for level-shifts from 2012 onward was employed to test whether those level-shifts were proportional to the prefecture-specific dose-rates derived from Cs-137 deposition in the 47 Japanese prefectures.

**Results:**

The overall trend of the low birth weight prevalence (LBWp) in Japan discloses a jump in 2012 with a jump odds ratio (OR) 1.020, 95%-confidence interval (1.003,1.037), *p*-value 0.0246. A logistic regression of LBWp on the additional dose-rate after the FDNPP accidents adjusted for prefecture-specific spatiotemporal base-line trends yields an OR per μSv/h of 1.098 (1.058, 1.139), *p*-value < 0.0001. Further adjusting the logistic regression for the annual population size and physician density of the prefectures, as well as for the counts of the dead, the missing, and the evacuees due to earthquake and tsunami (as surrogate measures for medical infrastructure and stress) yields an OR per μSv/h of 1.109 (1.032, 1.191), *p*-value 0.0046.

**Conclusions:**

This study shows increased low birth weight prevalence related to the Cs-137 deposition and the corresponding additional dose-rate in Japan from 2012 onward. Previous evidence suggesting compromised gestational development and pregnancy outcome under elevated environmental ionizing radiation exposure is corroborated.

## Introduction

Low birth weight (LBW) is defined as having a birth weight of < 2500 g. LBW is an objective and reliable indicator used as a comprehensive demographic reporting measure of fetal development and pregnancy outcome [1–4]. The OECD provides an international comparison of the LBW prevalence (LBWp), which shows that Japan is among the 5 countries with the highest LBWp in the range of 9 to 10% [5]. Environmental pollutants are consistently linked to untoward pregnancy outcome and reductions in birth weight [6–13]. LBW has been suggested as an indicator of genetic detriment caused by mutation in humans exposed to ionizing radiation [14]. Analyses of birth weight and duration of pregnancies in relation to maternal age, parity, and infant survival indicated that non-survivors were significantly lighter at birth than survivors [15]. LBW is closely linked to fetal and perinatal mortality and morbidity [16]. It has been reported to be associated with disorders in perinatal periods, in childhood, and in adulthood [17, 18]. Studies in Great Britain showed that people who had low birth weight were at increased risk of coronary heart disease and the disorders related to it [19]. Animal and human studies have shown that the LBW proportions increase with toxic exposure and with radiation exposure [20–22]. Smoking increases the LBWp in a dose-dependent manner [23], possibly due to elevated radionuclides in tobacco [24]. Females subject to pelvic radiotherapy experience an increased risk of pre-term delivery and LBW among their offspring [25]. Treatment of female childhood cancer patients may entail restricted fetal growth and pre-term births [26]. LBW was reported after dental radiography during pregnancy [27]. A cohort study in China identified multiple risk factors of LBW including radiation exposure of fathers [28]. A natural experiment in Taiwan revealed that prenatal exposure to a continuous low-dose radiation reduced the gestational length and increased the LBW proportion [29]. In Belarus, increased LBW prevalence was reported from the highly Chernobyl-contaminated regions Gomel and Mogilev [30]. In the Ukraine, detrimental radiation-dose dependent outcomes in neonates were observed [31]. Temporarily elevated LBWp was seen in Sweden after Chernobyl [32]. Detrimental effects in humans are supported by a recent animal study: In wild Japanese monkeys (*Macaca fuscata*), body weight growth rate and proportional head size were significantly lower in fetuses conceived after the Fukushima disaster [33].

Since several radiation inducible genetic effects, which can be associated with general radiation exposure were observed in all of Japan after the Fukushima nuclear power plant accidents [34–38], an increase in the LBW proportion in whole Japan was also conceivable. This argument is supported by the specific observation of increased thyroid cancer in children and adolescents in the Fukushima prefecture, which can be related to I-131 contamination in a dose-dependent manner [39–42]. Among the investigations after the Fukushima nuclear accidents, there are reports that LBW in humans is increasing and reports that deny the increase. In the following, we shortly address two reports that are questionnaire-based surveys with a response rate in the 50% range and one survey of a small number of births in one clinic in Fukushima [43–45]. Questionnaire-based studies are prone to selection bias and studies with small populations (mostly in clinical settings) may likely entail type-2 errors [46]. A questionnaire-based pregnancy and birth survey was conducted by the Radiation Medical Science Center for the Fukushima Health Management Survey [43]. In this study, an increase of the LBW proportion is documented in the combined two most eastern regions Iwaki and Soso compared to the five central and western regions of the Fukushima prefecture: OR 1.163, *p*-value 0.0723. This observation is further supported by an increase of the stillbirth proportion in Soso and Iwaki with OR 1.923, p-value 0.1321. Since this study [43] had a participation rate of below 60%, it is likely that significant effects would be obtained with lager populations considered during longer periods. Maternal and perinatal data (2008 to 2015) were retrospectively collected for singleton live births at a hospital located 23 km from the Fukushima nuclear power plant [44]. In 1101 births, LBWp was compared pre- and post-disaster. There was no increased LBWp in any year from 2011 onward. However, with 4 years before/after the accident, i.e., 140 births per year, which means about 10 LBW-births per year, it was unlikely to receive a meaningful result, i.e., there is a large type-2 error probability in this study [44]. A more recent investigation considered 12,804 maternal outcomes during 2011–2014 in the Fukushima Prefecture [45]. However, this study neither analyzed perinatal outcomes with distance from the nuclear accident nor chronological factors. Therefore, it is unclear whether increases of LBW are due to a temporary cause of the earthquake/tsunami or due to radiation exposure. These surveys cover the Fukushima Prefecture only incompletely over short periods. In the Miyagi Prefecture, the overall rate of LBW infants was reported to be 8.7%, which tended to be lower than LBWp in 2012 of 9.3 and 9.8% in 2013 [47]. In conclusion, the trends of the LBW prevalence in most of the Japanese prefectures have not yet been scrutinized, although detailed spatiotemporal data is publicly available.

In the present study, we analyzed data of the Japanese governments ‘Demographical Survey’, which accounts for all live births and all LBW children registered in Japan excluding births to parents living abroad. Therefore, not only Fukushima Prefecture but also the whole country with differently contaminated prefectures [48] was targeted, and statistical accuracy is guaranteed by using official practically complete long-term data from 1995 to 2018, i.e., 16 years (1995 to 2010) before and 7 years (2012 to 2018) after the nuclear power plant accidents in Fukushima in March 2011. Since radioactive contamination was much more comprehensively measured and documented in the prefecture Fukushima [49] compared to the rest of Japan [48], we put both measurement regimes in perspective. We propose a rescaling of the overall Japanese contamination measurements, and we study the possible association of the LBW prevalence with radioactivity at the prefecture level.

## Methods

### Vital statistics and psycho-social stressors

The Japanese Statistics Bureau publishes demographical information compiled by the Ministry of Health, Labor, and Welfare. Statistics include the annual numbers of live births and the annual counts of children with a low birth weight of < 2500 g (LBW), see Table 1 or the internet platform *Vital Statistics of Japan*. We investigated the spatiotemporal distribution of 26.158 million live births, of which 2.366 million births (9.04%) with weights < 2500 g, across 47 Japanese prefectures from 1995 to 2018. A considerable proportion of the Japanese population was physically and psychosocially affected to a significant degree by the Great East Japan Earthquake and subsequent tsunami [51]. Therefore, the counts of earthquake related deaths, the counts of the dead and missing after earthquake and tsunami, as well as the numbers of evacuees to and within any prefecture were obtained from official sources [52] and served as additional explicit ecological confounding variables in our logistic regression models. Since medical supply including medical information may also impact the general health-behavior and thus the prevalence of LBW, annual physician density by prefecture in Japan was deployed as a surrogate confounder variable in the spatiotemporal logistic regression models, see Table 2.

**Table 1:** Annual live births, live births with low birth weight (LBW: birth weight < 2500 g), and LBW proportions (LBWp) in Japan stratified by exposure status of prefectures; see Table 3 and Fig. 2; Table 1 excludes 3774 live births (including 197 LBWs, 5.2%) to Japanese parents in foreign countries; see https://www.mhlw.go.jp/english/database/db-hw/vs01.html

Year	5 highly contaminated prefectures Fukushima, Miyagi, Ibaraki, Tochigi, Iwate			5 moderately contaminated prefectures Yamagata, Saitama, Tokyo, Kanagawa, Chiba			37 slightly contaminated prefectures with ISO codes 1, 2, 5, 10, 15 to 47			Total		
	live births	LBW	LBWp	live births	LBW	LBWp	live births	LBW	LBWp	live births	LBW	LBWp
**1995**	103,490	7869	0.0760	311,160	23,216	0.0746	772,147	58,016	0.0751	1,186,797	89,101	0.0751
**1996**	104,322	7550	0.0724	315,799	23,712	0.0751	786,132	59,611	0.0758	1,206,253	90,873	0.0753
**1997**	102,020	7899	0.0774	312,979	24,415	0.0780	776,360	61,502	0.0792	1,191,359	93,816	0.0787
**1998**	103,271	8165	0.0791	315,199	25,278	0.0802	784,388	64,157	0.0818	1,202,858	97,600	0.0811
**1999**	101,549	8349	0.0822	310,282	25,817	0.0832	765,596	64,989	0.0849	1,177,427	99,155	0.0842
**2000**	102,092	8825	0.0864	315,728	27,073	0.0857	772,517	66,980	0.0867	1,190,337	102,878	0.0864
**2001**	100,806	8672	0.0860	311,095	26,595	0.0855	758,563	67,600	0.0891	1,170,464	102,867	0.0879
**2002**	98,515	8614	0.0874	311,474	28,049	0.0901	743,671	67,641	0.0910	1,153,660	104,304	0.0904
**2003**	95,674	8576	0.0896	304,896	27,602	0.0905	722,870	66,133	0.0915	1,123,440	102,311	0.0911
**2004**	93,692	8608	0.0919	303,562	28,218	0.0930	713,291	67,995	0.0953	1,110,545	104,821	0.0944
**2005**	89,016	8258	0.0928	292,414	27,402	0.0937	680,930	65,601	0.0963	1,062,360	101,261	0.0953
**2006**	90,578	8495	0.0938	303,268	28,632	0.0944	698,652	67,421	0.0965	1,092,498	104,548	0.0957
**2007**	89,317	8528	0.0955	304,808	28,714	0.0942	695,533	67,913	0.0976	1,089,658	105,155	0.0965
**2008**	88,826	8394	0.0945	307,184	29,090	0.0947	694,973	66,986	0.0964	1,090,983	104,470	0.0958
**2009**	86,431	8053	0.0932	304,949	28,711	0.0942	678,556	65,905	0.0971	1,069,936	102,669	0.0960
**2010**	85,459	8214	0.0961	305,933	28,864	0.0943	679,787	65,960	0.0970	1,071,179	103,038	0.0962
**2011**	81,576	7808	0.0957	299,020	28,129	0.0941	670,088	64,433	0.0962	1,050,684	100,370	0.0955
**2012**	80,622	7885	0.0978	296,914	28,026	0.0944	659,628	63,399	0.0961	1,037,164	99,310	0.0958
**2013**	80,672	8087	0.1002	298,278	28,081	0.0941	650,812	62,454	0.0960	1,029,762	98,622	0.0958
**2014**	78,704	7643	0.0971	294,105	27,341	0.0930	630,665	60,778	0.0964	1,003,474	95,762	0.0954
**2015**	78,014	7543	0.0967	297,591	27,429	0.0922	630,019	60,229	0.0956	1,005,624	95,201	0.0947
**2016**	74,931	7280	0.0972	289,991	26,874	0.0927	611,991	57,925	0.0947	976,913	92,079	0.0943
**2017**	72,500	6989	0.0964	281,503	25,974	0.0923	592,011	56,388	0.0952	946,014	89,351	0.0944
**2018**	69,184	6656	0.0962	275,332	25,345	0.0921	573,845	54,266	0.0946	918,361	86,267	0.0939
**Total**	2,151,261	192,960	0.0897	7,263,464	648,587	0.0893	16,743,025	1,524,282	0.0910	26,157,750	2,365,829	0.0904

**Table 2:** Mean annual population (1000) for the Japanese prefectures 1995 to 2018, dead and missing after earthquake and tsunami 2011, earth-quake related deaths, evacuated persons within or to the prefectures (sources: National Police Agency March 8, 2019; Reconstruction Agency September 30, 2018), mean annual live births, mean annual LBW and LBWp, jump OR in LBWp from 2012 onward, and 95%-CI for jump OR in LBWp from 2012 to 2018; see https://stats-japan.com/t/kiji/10343

Prefecture	ISO code	mean annual population (1000) 1995–2018	pyhsician density per 1000 population	dead and missing after earthquake and tsunami	earth-quake related deaths	evacuated persons within or to prefecture	mean annual live births 1995–2018	mean annual LBW 1995–2018	mean annual LBWp 1995–2018	jump OR in LBWp from 2012 onward	95%-CI for jump OR in LBWp
**Hokkaido**	1	5557.8	2.5	1	0	3003	42,115.3	3880.6	0.092	1.046	(1.015, 1.079)
**Aomori**	2	1403.0	2.1	4	0	1410	10,842.5	924.6	0.085	0.953	(0.897, 1.014)
**Iwate**	3	1355.5	2.1	5788	467	42,716	10,560.7	915.3	0.087	1.047	(0.985, 1.113)
**Miyagi**	4	2345.8	2.4	10,761	928	127,825	19,827.2	1724.0	0.087	1.059	(1.013, 1.106)
**Akita**	5	1118.3	2.4	0	0	1473	7571.4	672.0	0.089	1.014	(0.944, 1.088)
**Yamagata**	6	1193.1	2.3	2	2	13,538	9398.9	758.0	0.081	1.094	(1.024, 1.170)
**Fukushima**	7	2041.9	2.1	1810	2250	98,595	17,277.0	1507.3	0.087	1.078	(1.027, 1.131)
**Ibaraki**	8	2958.6	1.9	25	42	6077	24,918.3	2234.0	0.090	1.049	(1.009, 1.092)
**Tochigi**	9	1996.8	2.3	4	0	3157	17,052.7	1659.5	0.097	1.083	(1.035, 1.133)
**Gunma**	10	2005.1	2.4	1	0	1974	16,868.0	1511.8	0.090	1.003	(0.956, 1.051)
**Saitama**	11	7080.9	1.7	0	1	4778	60,963.0	5470.0	0.090	1.024	(0.998, 1.050)
**Chiba**	12	6073.4	2.0	23	4	3608	51,154.6	4418.6	0.086	1.025	(0.996, 1.054)
**Tokyo**	13	12,716.8	3.2	7	1	9505	103,513.0	9304.9	0.090	1.011	(0.991, 1.031)
**Kanagawa**	14	8799.5	2.1	4	3	2888	77,614.8	7073.0	0.091	1.017	(0.994, 1.040)
**Niigata**	15	2402.7	2.1	0	0	6990	19,002.3	1610.8	0.085	1.072	(1.024, 1.122)
**Toyama**	16	1099.7	2.6	0	0	377	8806.5	744.5	0.085	0.976	(0.912, 1.044)
**Ishikawa**	17	1170.4	3.0	0	0	499	10,197.8	860.6	0.084	1.122	(1.054, 1.195)
**Fukui**	18	811.7	2.6	0	0	443	7179.9	580.9	0.081	1.030	(0.955, 1.110)
**Yamanashi**	19	867.6	2.4	0	0	837	7191.0	709.4	0.099	0.965	(0.900, 1.035)
**Nagano**	20	2166.2	2.4	0	3	1363	18,379.8	1631.5	0.089	0.928	(0.886, 0.971)
**Gifu**	21	2081.7	2.2	0	0	412	17,751.2	1562.4	0.088	0.984	(0.939, 1.031)
**Shizuoka**	22	3752.0	2.1	0	0	1399	32,300.2	3140.3	0.097	1.016	(0.983, 1.051)
**Aichi**	23	7255.8	2.2	0	0	1260	69,496.4	6419.0	0.092	1.015	(0.992, 1.040)
**Mie**	24	1847.2	2.3	0	0	413	15,698.8	1361.5	0.087	1.064	(1.012, 1.118)
**Shiga**	25	1376.1	2.3	0	0	385	13,247.5	1150.5	0.087	1.006	(0.954, 1.061)
**Kyoto**	26	2629.8	3.4	0	0	1056	21,623.1	1961.3	0.091	0.968	(0.929, 1.009)
**Osaka**	27	8822.0	2.8	0	0	1335	78,362.3	7110.8	0.091	0.979	(0.957, 1.002)
**Hyogo**	28	5535.6	2.5	0	0	1033	48,658.1	4396.1	0.090	0.982	(0.955, 1.010)
**Nara**	29	1408.2	2.5	0	0	166	11,420.2	992.0	0.087	0.973	(0.918, 1.031)
**Wakayama**	30	1022.1	3.0	0	0	130	8150.3	711.9	0.087	0.973	(0.908, 1.042)
**Tottori**	31	596.6	3.2	0	0	194	5064.3	457.7	0.090	1.136	(1.043, 1.237)
**Shimane**	32	732.1	2.9	0	0	142	5926.8	558.5	0.094	1.013	(0.938, 1.095)
**Okayama**	33	1943.4	3.1	0	0	771	17,223.8	1492.0	0.087	1.091	(1.040, 1.144)
**Hiroshima**	34	2864.9	2.7	0	0	539	25,553.9	2323.4	0.091	1.021	(0.983, 1.060)
**Yamaguchi**	35	1475.8	2.6	0	0	206	11,667.3	1082.1	0.093	1.013	(0.958, 1.071)
**Tokushima**	36	796.6	3.3	0	0	100	6266.2	523.8	0.084	1.018	(0.941, 1.102)
**Kagawa**	37	1003.7	2.9	0	0	111	8648.0	745.5	0.086	1.026	(0.959, 1.097)
**Ehime**	38	1448.4	2.7	0	0	232	11,803.3	1014.8	0.086	1.007	(0.951, 1.066)
**Kochi**	39	777.2	3.2	0	0	142	5879.8	574.7	0.098	0.977	(0.905, 1.055)
**Fukuoka**	40	5047.1	3.1	0	0	744	45,845.4	4393.5	0.096	1.015	(0.987, 1.044)
**Saga**	41	859.1	2.9	0	0	304	7816.5	702.0	0.090	1.046	(0.977, 1.121)
**Nagasaki**	42	1457.0	3.1	0	0	174	12,580.7	1092.7	0.087	1.012	(0.957, 1.069)
**Kumamoto**	43	1827.9	3.0	0	0	312	16,323.8	1457.8	0.089	0.993	(0.947, 1.042)
**Oita**	44	1200.7	2.8	0	0	350	10,077.7	893.2	0.089	1.038	(0.977, 1.103)
**Miyazaki**	45	1143.0	2.5	0	0	260	10,252.3	983.2	0.096	1.003	(0.946, 1.062)
**Kagoshima**	46	1727.6	2.7	0	0	281	15,164.8	1481.5	0.098	1.039	(0.992, 1.090)
**Okinawa**	47	1366.8	2.5	0	0	970	16,669.2	1803.2	0.108	1.093	(1.047, 1.142)

### Cs-137 deposition

**Fig. 1:**
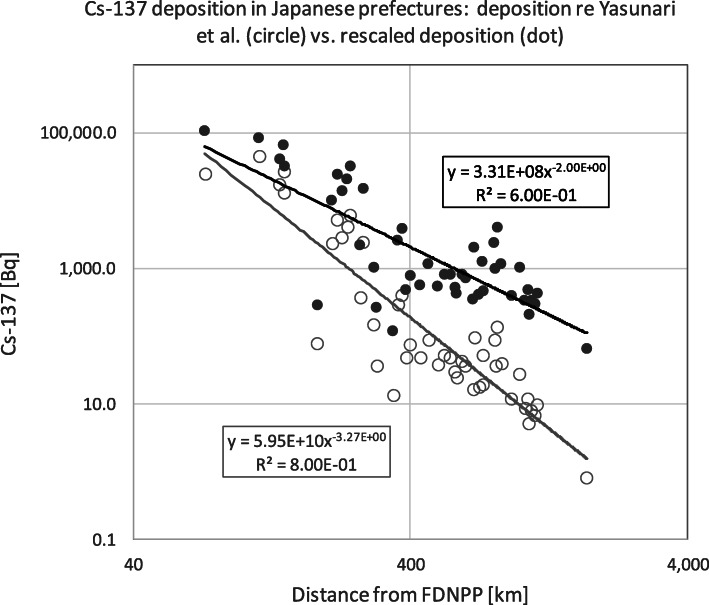
Deposition of Cs-137 in the 47 Japanese prefectures according to Yasunari et al. [48] by the prefectures’ distances from the Fukushima Daichi Nuclear Power Plant (FDNPP); gray circles: original Yasunari et al. data; black dots: deposition for Fukushima corrected and remaining depositions rescaled to a decay of r^−2^ with distance r, see Table 3

Yasunari et al. published average prefecture-specific Cs-137 deposition after the Fukushima nuclear power plant accidents for the 47 prefectures of Japan [48]; see Table 3. Yasunari et al. assumed that half of the total cesium deposited was Cs-134. Therefore, it is easily possible to re-scale all calculations in this paper to total cesium in place of Cs-137. The Yasunari et al. data understate the true Cs-137 deposition, which underestimation may be supported by the following aspects:

- Yasunari et al.’s data based on measurements restricted to March 20th to April 19th, 2011.
- Yasunari et al. report a value of 24.7 kBq/m^2^ Cs-137 for Fukushima prefecture (see Table 3), whereas the UNSCEAR data set 2013/2014 documents a mean value of 153.957 kBq/m^2^ Cs-137, which amounts to a factor 6 underestimation of the deposition in Fukushima by Yasunari et al. [48], see the Excel-file provided by UNSCEAR as referenced in Table 3 [49].
- It is implausible that Fukushima prefecture would be less contaminated than Miyagi and Ibaraki prefectures, see Table 3.
- The Yasunari et al. data decay with r^-3.27^ at distance r form the FDNPP, see Fig. 1, whereas a theoretical decay law of r^-1.42^ is expected according to UNSCEAR [50], and as empirically confirmed for the Fukushima prefecture [40].

**Table 3:** Distances of the centers of the Japanese prefectures’ area polygons from the FDNPP, Cs-137 deposition in the Japanese prefectures after the Fukushima nuclear power plant accidents as of March 2011 according to [48], rescaled Cs-137 deposition according to [48–50], and dose-rate [μSv/h] derived from the rescaled deposition; see https://www.unscear.org/docs/publications/2013/UNSCEAR_2013_Annex-A_Attach_C-2.xls and http://www.pnas.org/content/108/49/19530.full

Prefecture	ISO code	distance from FDNPP [km]	Cs-137 [Bq/m^**2**^] Yasunari et al.	Cs-137 [Bq/m^**2**^] rescaled	μSv/h
**Fukushima**	7	72.4	24,718.4	^a^106867.0	0.8696
**Miyagi**	4	113.8	44,696.6	83,416.9	0.6903
**Ibaraki**	8	139.7	26,368.9	65,259.1	0.5513
**Tochigi**	9	134.9	17,380.7	40,982.0	0.3651
**Iwate**	3	242.6	6022.7	31,908.4	0.2954
**Yamagata**	6	140.5	12,755.5	31,826.5	0.2948
**Saitama**	11	217.9	5256.1	24,011.4	0.2347
**Tokyo**	13	237.1	4063.0	20,854.3	0.2105
**Kanagawa**	14	270.1	2435.5	14,962.8	0.1652
**Chiba**	12	226.0	2878.0	13,826.4	0.1564
**Gunma**	10	208.6	2317.9	9971.1	0.1268
**Kochi**	39	824.6	137.0	3921.2	0.0802
**Aomori**	2	373.7	401.7	3860.4	0.0797
**Shizuoka**	22	360.0	284.7	2598.8	0.0700
**Hiroshima**	34	805.1	87.0	2409.4	0.0686
**Akita**	5	263.9	366.6	2180.4	0.0668
**Tottori**	31	681.3	93.1	2047.9	0.0658
**Tokushima**	36	727.7	51.0	1228.6	0.0595
**Ehime**	38	852.5	38.4	1150.7	0.0589
**Fukui**	18	464.1	88.3	1144.0	0.0588
**Oita**	44	990.4	27.7	1020.7	0.0579
**Yamanashi**	19	295.1	146.8	1018.7	0.0578
**Shimane**	32	810.6	35.7	998.0	0.0577
**Kyoto**	26	556.7	48.4	805.9	0.0562
**Hyogo**	28	614.8	41.6	794.2	0.0561
**Mie**	24	529.8	51.0	793.0	0.0561
**Gifu**	21	399.2	73.8	776.9	0.0560
**Wakayama**	30	633.8	36.1	718.8	0.0555
**Aichi**	23	433.3	48.1	566.9	0.0544
**Shiga**	25	503.0	37.1	537.1	0.0541
**Nara**	29	579.5	29.2	513.9	0.0540
**Ishikawa**	17	386.6	48.1	484.5	0.0537
**Miyazaki**	45	1061.8	11.7	474.5	0.0537
**Kagawa**	37	730.6	18.9	457.8	0.0535
**Kagoshima**	46	1150.0	9.5	430.1	0.0533
**Osaka**	27	586.9	24.0	429.8	0.0533
**Okayama**	33	706.9	17.5	405.0	0.0531
**Yamaguchi**	35	928.0	11.8	397.5	0.0531
**Hokkaido**	1	674.5	16.0	347.1	0.0527
**Saga**	41	1092.6	7.9	333.3	0.0526
**Fukuoka**	40	1034.8	8.5	332.7	0.0526
**Nagasaki**	42	1126.0	6.8	299.1	0.0523
**Niigata**	15	184.2	77.2	279.7	0.0522
**Nagano**	20	302.8	36.7	263.9	0.0520
**Kumamoto**	43	1070.2	5.1	209.1	0.0516
**Toyama**	16	346.3	13.4	116.0	0.0509
**Okinawa**	47	1727.5	0.8	63.5	0.0505

^a^according to *n* = 2160 locations with Cs-137 Bq < 2.0E+ 6 in the Excel file of reference [49];

**Fig. 2:**
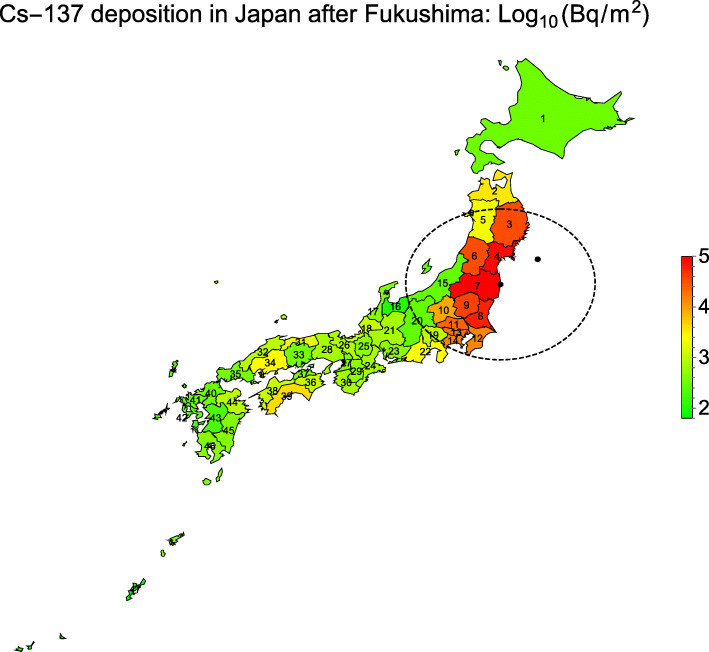
Geographic region value plot of the decadic logarithm for the rescaled Cs-137 deposition in 47 Japanese prefectures after the Fukushima nuclear power plant accidents as of March 2011 [48], see Table 3; indication of the positions of the earthquake epi-center, the FDNPP, and a 300 km geo-circle around FDNPP; for the prefecture codes see Table 2 or Table 3

Since strong underestimation of radiation exposure would exaggerate any dose-specific radiation risk estimates, we suggest and propagate a correction and a rescaling of the Yasunari et al. deposition data for all of Japan despite the disadvantages of these data listed above. The rationale behind this is that the Yasunari et al. data, while restricted to a narrow time frame nevertheless reflect a valid mutual relative exposure status amongst the prefectures. To this end, we firstly increased the original deposition value 24,718.4 Bq/m^2^ of the Fukushima prefecture by a factor of 4.3 to 106,867.0 Bq/m^2^ based on the MEXT/UNSCEAR data [49] excluding 20 locations in the immediate vicinity of FDNPP with more than 2.0E+ 6 Bq/m^2^ with less likely importance for public exposure. Secondly, we rescaled the deposition data with the original decay-rate r^-3.27^ to a decay of r^-2.00^, which is a compromise between the theoretical decay r^-1.42^ by UNSCEAR [50] and the Yasunari decay r^-3.27^. The rescaling details and results are depicted in Fig. 1 and listed in Table 3. Figure 2 shows a geographic region value plot for the rescaled Cs-137 deposition in the Japanese prefectures.

### Dose-rate (μSv/h) derived from Cs-137 deposition

**Fig. 3:**
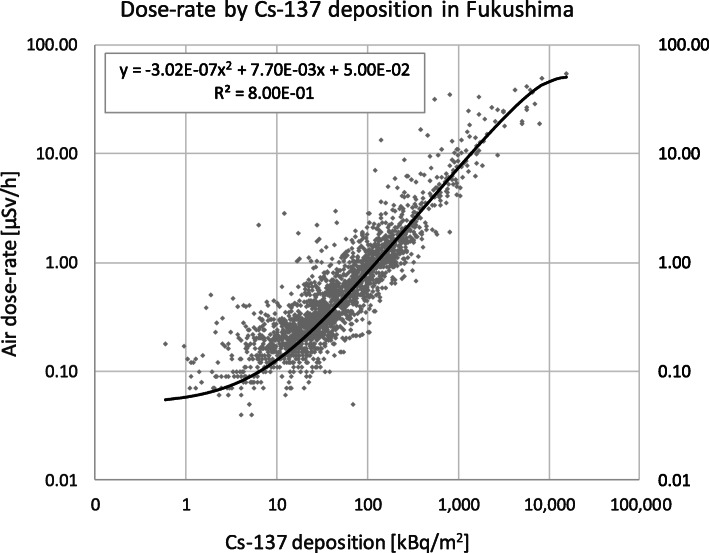
Association of the dose-rate [μSv/h] at 1 m height with the Cs-137 deposition [kBq/m^2^] in and near the Fukushima prefecture for 2180 positive deposition measurements and 2175 positive dose-rate readings**;** see [49] and Fig. 5A in [40] https://www.unscear.org/docs/publications/2013/UNSCEAR_2013_Annex-A_Attach_C-2.xls

The United Nations Scientific Committee on the Effects of Atomic Radiation (UNSCEAR) published Cs-137 deposition and corresponding dose-rate readings at 2180 locations in the Fukushima prefecture or close to the borders of the Fukushima prefecture [49]. These data were provided by the Government of Japan as described in the report titled ‘Summarized version of the results of the research on distribution of radioactive substances discharged by the accident at TEPCO’s Fukushima Daiichi NPP’. The Japan Atomic Energy Authority (JAEA) conducted the survey with cooperation of universities and research institutes. The Ministry of Education, Culture, Sports, Science, and Technology in Japan (MEXT) was responsible for the measurements and their validity. UNSCEAR reviewed and published the dataset [49]. The single dose-rate readings resulting from all relevant deposited radionuclides including Cs-134 range from 0.040 μSv/h to 54.800 μSv/h, with mean 1.259 μSv/h and median 0.40 μSv/h. The single C-137 measurements range from 590 Bq/m^2^ to 15,450,928 Bq/m^2^, with mean 153,957 Bq/m^2^ and median 39,714 Bq/m^2^. A 2^nd^ degree regression of the dose-rate on the Cs-137 deposition allows the translation of fallout to dose-rate. The functional details of this association are presented in Fig. 3 and the resulting dose-rates are listed in Table 3. Unfortunately, fine-resolution contamination data as available for the Fukushima prefecture, does not exist for all of Japan.

### Statistical methods

A powerful method to assess data that contains spatial as well as temporal information is spatiotemporal (logistic) regression [38, 53–57]. The basic idea is to adjust a regression model for region-specific trend functions and to allow for local or global level-shifts at certain points in time, or, preferably, to allow for the heights of local drops or jumps to be proportional to the contamination level of the regional strata. The advantage of this spatiotemporal method is optimization of adjustment and minimization of confounding by considering partial trends of regional units. Values of the outcome variable (here LBW) in those partial trends are modeled and compared within the same regional stratum, as the target variable describing the characteristic of interest varies from year to year. Information on several regional units is combined in a global spatiotemporal model, giving rise to tests of local or global change-points in time as well as spatial trends in the outcome variable with regionally determined contamination or exposure. In the present study, we considered the 47 prefectures of Japan with their documented individual annual LBW proportions (1995 to 2018), their original [48] and rescaled Cs-137 depositions, and the associated dose-rates compiled in Table 3. We tested whether possible changes of LBWp from 2012 onward were dependent on Cs-137 fallout at the prefecture level. We further adjusted the spatiotemporal logistic regression model for the annual population size of the prefectures (1995 to 2015), for the number of physicians per 1000 population, as well as for the counts of the dead, the missing, and the evacuees due to the earthquake and the tsunami as surrogate measures for the available prefecture-specific medical infrastructure and the stress associated with this triple catastrophe and its aftermath, see Table 2. For data processing, statistical analyses, and results display, we used Microsoft Excel 2016 (Office 365), R 3.5.1 (Version 2017-10-04), Wolfram MATHEMATICA 11.3, and mostly SAS/STAT software 9.4 (SAS Institute Inc.: SAS/STAT User’s Guide, Version 9.4, Cary NC: SAS Institute Inc.,© 2002–2012).

## Results

**Fig. 4:**
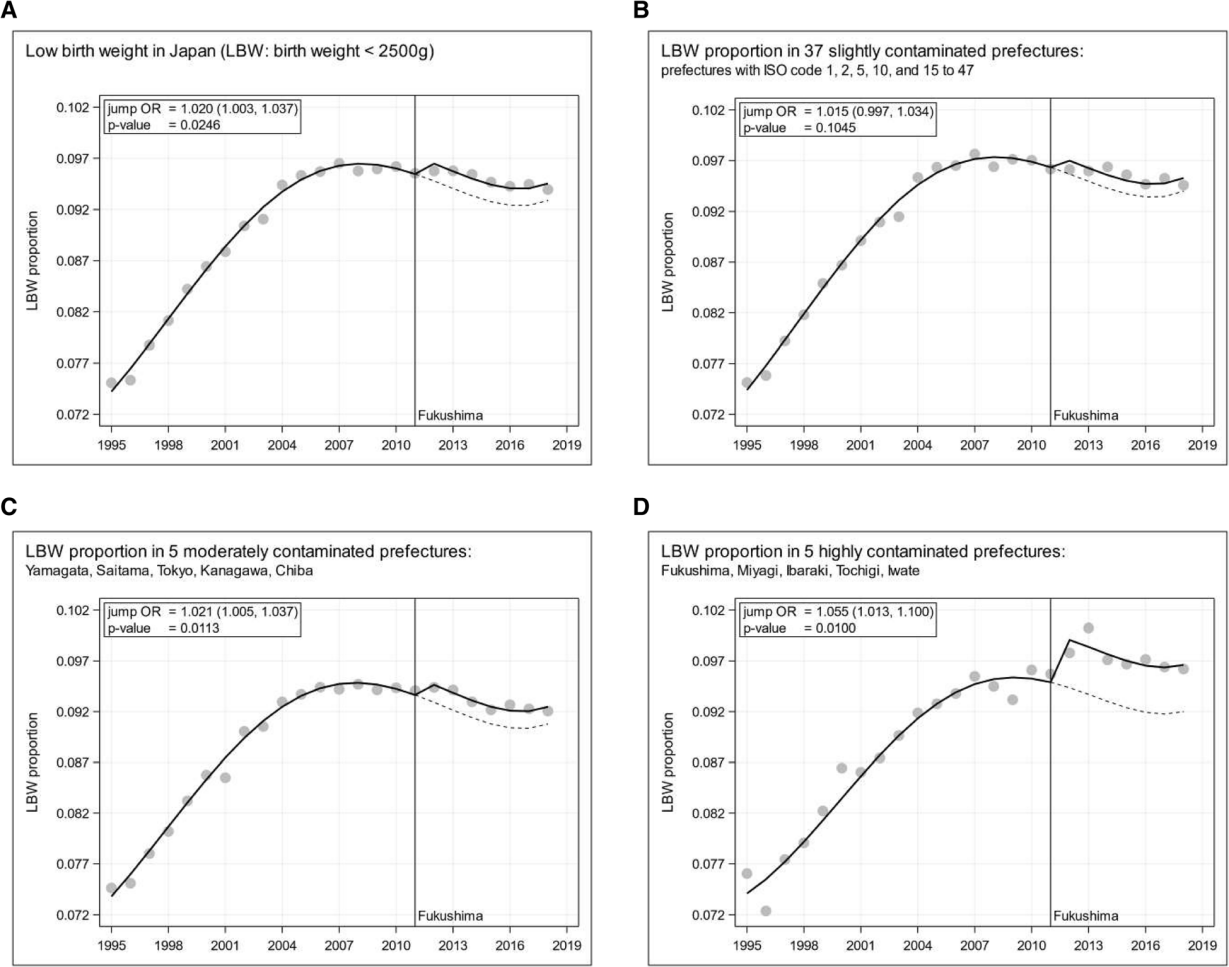
Low birth weight (LBW) proportion in Japan 1995 to 2018; 4^th^ degree polynomial logistic regression trends allowing for jumps from 2012 onward; **A**: Japan; **B**: Japan excluding 10 exposed prefectures; **C**: 5 moderately exposed prefectures; **D**: 5 highly exposed prefectures, see Table 1 for the absolute counts, the relative frequencies, and the ISO codes of the prefectures

Figure 4A shows the annual marginal LBW distribution for all of Japan 1995 to 2018, excluding the births to Japanese parents in foreign countries (number of births abroad: 3774, LBWp = 0.052). See the columns ‘Total’ in Table 1 for the corresponding absolute counts and the LBW proportions (LBWp). As a first step, we fit to this overall LBWp a smooth 4^th^ degree polynomial allowing for a change-point in 2012 after the Fukushima nuclear power plant accidents. This approach discloses a significant jump in 2012 with a jump odds ratio (OR) 1.020, 95%-CI (1.003, 1.037), *p*-value 0.0246, see Fig. 4A. In Japan, people consider that the reportedly healthy lean structure of women led to reduced weight gain during pregnancy and contributed to the rise in LBW prior to 2007, because low BMI and poor weight gain are risk factors for LBW. However, the increase in LBWp reached its overall maximum in 2007 (see Fig. 4A), and subsequently LBWp slightly fell or remained nearly constant due to a change in health-awareness and behavior [58].

Considering the possibility that the jump height in 2012 may be associated with the Cs-137 fallout in the prefectures, we analyze and depict in a second step the behavior of LBWp in the 3 strata of prefectures according to Table 1: in the 37 least contaminated prefectures, in the 5 moderately contaminated prefectures (Yamagata, Saitama, Tokyo, Kanagawa, Chiba), and in the 5 most contaminated prefectures (Fukushima, Miyagi, Ibaraki, Tochigi, Iwate). Figures 4B-D display the result: the higher the fallout in the prefectures, the higher the jumps in LBWp from 2012 to 2018. The excess LBW counts and their respective 95%-confidence intervals corresponding to the jump odds ratios (ORs) in the LBWp trends in Fig. 4A-D are (A) 11,561 (1470, 21,793), (B) 5659 (− 1165, 12,585), (C) 3458 (778, 6172), and (D) 2484 (584, 4448), respectively.

**Fig. 5:**
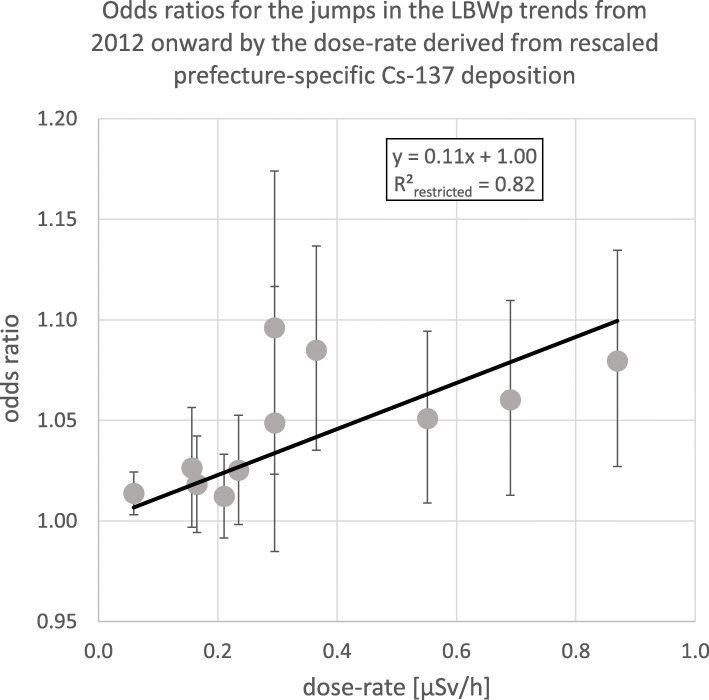
Odds ratios for the jumps in the low birth weight proportion trends (LBWp) from 2012 onward by prefecture-specific dose-rates derived from the rescaled Cs-137 deposition in the Japanese prefectures from March 20th to April 19th 2011; restricted linear regression yields trend *p*-value < 0.0001; the left data point summarizes and represents 37 slightly radiologically impacted prefectures, the 10 data points from the right represent the 10 prefectures with high to moderate pollution, see Table 1

Table 2 and Fig. 5 generalize and visualize the effects seen in Fig. 4 by listing and plotting the prefecture-specific level-shifts in 2012 in the LBWp trends against the average dose-rate in the prefectures. The combination of the 37 least contaminated prefectures in one group avoids an overly scattered picture for these regions in the left part of Fig. 5. The leftmost data point in Fig. 5 represents this group of the 37 low or only slightly contaminated prefectures. In Fig. 5, a variance weighted straight line regression of the individual jump odds ratios against the dose-rates discloses a significant linear relationship (R^2^ = 0.82) with slope 0.11 per μSv/h and *p*-value < 0.0001.

A more direct approach is logistic regression of LBWp on the additional dose-rate after Fukushima adjusted for prefecture-specific spatiotemporal base-line trends [57]. This yields an OR per μSv/h of 1.098 (1.058, 1.139), *p*-value < 0.0001. By additionally adjusting this spatiotemporal logistic regression for the counts of the earthquake-related deaths, the dead and the missing after the tsunami, and the counts of evacuees within and to the prefectures as surrogate measures for the disaster-related stress from 2011 onward (see Table 2), as well as additionally taking into account the prefecture-specific population size and physician density per 1000 population as surrogate measures of general stress and available medical infrastructure, we obtain a somewhat larger, however less precise adjusted OR per μSv/h of 1.109 (1.032, 1.191), p-value 0.0046. The decreased precision resulting from this additional adjustment may be explained by variance inflation in the spatiotemporal logistic regression model due to partly correlated surrogate confounder measures. In summary, the increase of the background dose-rate by 1 μSv/h elevates the prevalence odds of low birth weight babies by approximately 10%. Note, 1 μSv/h translates to a dose of 8.8 mSv/year. Importantly, without the suggested rescaling of the Yasunari et al. exposure data, the dose-rate specific effect would be 50% in place of 10%, and this would likely be an overestimation of the radiation effect due to an obvious underestimation of the overall Cs-137 deposition and the associated dose-rate across Japan by Yasunari et al. [48].

**Fig. 6:**
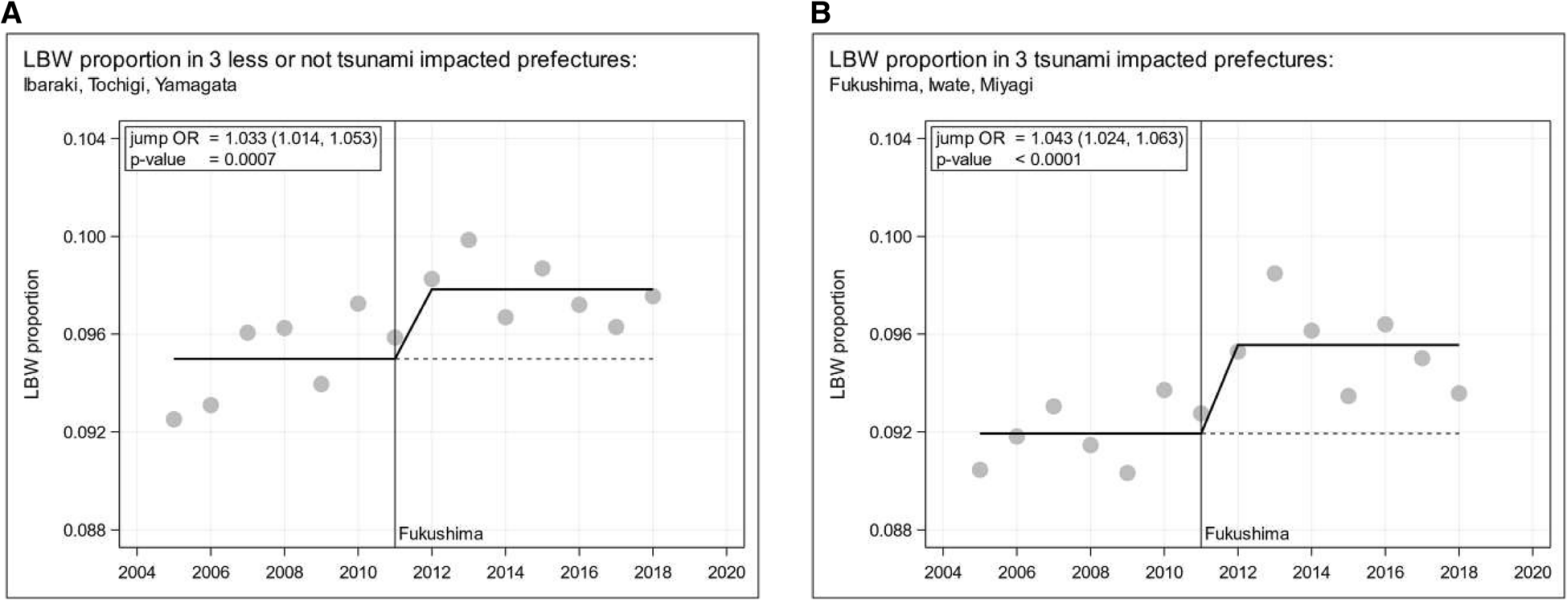
Low birth weight (LBW) proportion and parsimonious constant trends allowing for jumps in 2012 in 6 Japanese Fukushima exposed prefectures (2005 to 2018) stratified by tsunami impact; **A**: low tsunami impact in Ibaraki, Tochigi, and Yamagata; **B**: high tsunami impact in Fukushima, Iwate, and Miyagi

To more directly assess and display the relative impacts of the earthquake and the tsunami versus the effects of the Cs-137 deposition and the associated dose-rate on LBW, we compared the three contaminated prefectures Fukushima, Iwate, and Miyagi, where the dead and missing persons due to earthquake and tsunami were numerous (*n* = 18,359), to the somewhat weaker contaminated Ibaraki, Tochigi, and Yamagata where only relatively few immediate deaths occurred and few persons were missing (*n* = 31), see Table 2. Figure 6A and B show that for the less versus strongly earthquake and tsunami impacted groups of prefectures, the LBWp jump heights are similar with largely overlapping 95%-CIs and with similar *p*-values. Therefore, the long-term increasing LBWp is essentially independent of the direct or protracted impact of earthquake and tsunami.

## Discussion

This study strengthens the evidence provided by previous investigations [20, 26–30, 32] that elevated exposure to ionizing radiation increases the prevalence of low birth weight children. The proportion of low birth weight babies in Japan (LBW < 2500 g) was increasing continuously from 1995 to a peak value in 2007, see Fig. 4A. Control of maternal weight gain in reproductive-age women in the general Japanese population appeared to be a major factor involved in the increase in LBW babies before 2007 [4]. Since 2007, the conventional practice of ‘*suppressing weight gain during pregnancy to within 10 kg*’ has been changed to not reducing the weight gain too much. This may be the reason why most of the prefectures have stopped increasing LBW since peaking around 2007. The revision may spread rapidly in prefectures with large cities and high physician density, and, therefore, the degree of spread may vary between the prefectures. This situation suggests the adjustment of the spatiotemporal LBWp logistic regression models not only for its estimable and known spatiotemporal base-line trend parameters and possible determinants, such as Cs-137 deposition and earthquake-related stress measures, but also for the annual population size and the physician density of the prefectures. In Table 2 we compiled these potential ecological confounders. However, the adjustment for the additional confounders (population counts, physician density, and the triple-disaster-related stress indicators from 2012 onward) does not change our effect estimate of approximately 10% per 1 μSv/h. The reason for this may be that all major prefecture-specific information is already captured by the prefecture-specific spatiotemporal base-line trends accounted for in the adopted spatiotemporal regression approach [57].

Our investigation disclosed a positive association between the Cs-137 deposition across Japan after Fukushima with the prevalence of low birth weight (< 2500 g). Therefore, previously reported epidemiological health detriment after Fukushima [34–37, 39–41, 59] can be generalized and corroborated. Nevertheless, the question whether ionizing radiation exposure of young people in reproductive age or peri-conceptional and embryonic radiation exposure impair fetal and post-natal development of offspring remains a controversial issue. There are articles in favor of and against this hypothesis, e.g., [60, 61]. A problem with statistically negative studies in the clinical setting is sample size - typically in the range of a few thousand [61] or less. Small sample sizes generally entail low statistical power implying large type-2 error probabilities. It may be rather improbable to detect relevant changes in low birth weight proportions, say in the order of 10%, with population sizes ranging in the thousands only. For example, a two-sided one-sample binomial test for testing a hypothetically increased LBWp of 0.11 against a typical null-LBWp of 0.10 (i.e. 10% increase) requires a sample size of 7248 to achieve a statistical power of at least 80%. For more realistic two-sample scenarios involving additional independent LBW-determinants entailing enhanced biological variability, the required sample sizes for obtaining meaningful results would be even larger. Therefore, it is of no surprise that no unequivocal evidence has been obtained yet. For example, in a study mentioned in the introduction [31], there was no statistically significant effect (*p*-value > 0.1) of fetal dose on birth weight in 2582 in-utero-exposed individuals from northern Ukraine for whom estimates of fetal thyroid I-131 dose were available. Because of this relatively small population size (*n* = 2582), the statistical power for detecting a relevant 10% increase in LBW prevalence, which prevalence is itself in the range of 10%, achieves only 40%. In contrast, our study with effective sample sizes in the order of 2,000,000 live births in the 5 moderately contaminated prefectures and 500,000 live births in the 5 highly contaminated prefectures from 2012 to 2018 (see Table 1) yields statistical powers of over 80% for detecting 2 and 4% increases in the LBW prevalence, after Fukushima, respectively.

Under the headline “*Radiation-induced mutation rates in man*”, UNSCEAR [14] emphasized already in the year 1958 “*All the results obtained are subject to an inevitable sampling error which necessitates the collection of a very large amount of data. A number of quantitative characters, such as birth weight, size and various anthropometric measurements, as well as statistical data, such as neo-natal mortality, have been suggested and examined. Unfortunately, the precise genetic component in these variables is not known; on the contrary, they are known to be dependent upon factors which are economic (standard of living), demographic (age of parents, order of birth, etc.) and sociological (medical care)*.” The sample size issue addressed in this statement may be resolved when instead of at most thousands of births in clinical settings many millions of births in ecological epidemiological studies can be considered: After the nuclear accidents of Chernobyl and Fukushima, the populations of large regions or even whole countries have been exposed to additional ionizing radiation significantly elevating the existing background radiation by, e.g., 10% or above [54, 56, 62, 63]. Moreover, in a large-scale ecological design, as the one presented here, the socio-demographic and environmental determinants of the low birth weight prevalence can be considered similar in and comparable between the regional units (prefectures). The differences within and between the regional trends from 2012 onward can be assessed by spatiotemporal logistic regression adjusted for appropriately chosen base-line trend parameters and further LBW determinants and ecological confounders [57].

## Conclusions

This study shows increased low birth weight prevalence across Japan related to the prefecture-specific dose-rate derived from Cs-137 deposition after Fukushima. One (1.0) μSv/h (equivalent to 8.8 mSv/year) increases the odds of observing low birth weight events by approximately 10%. Therefore, previous investigations suggesting compromised gestational development and impaired pregnancy outcome under elevated ionizing radiation levels have been corroborated by the present study. These findings, in the overall view, call for intensifying bio-physical research in exposure mechanisms and exposure pathways of natural or artificial ionizing radiation. Biological, epidemiological, and medical research should aim at clarifying the genetic and the carcinogenic consequences of enhanced radiation in the environment or in the workplace. Radiation-induced genetic effects may occur without immediately obvious link to spectacular incidents or accidents [63, 64]. Therefore, the legislator, the nuclear industry, and the nuclear and radio-pharmaceutical medicine must impose and exert even greater care when processing, employing, and disposing radioactive materials.

## Data Availability

The employed data has exclusively been published previously and/or it is contained in the Tables and in the Figures included in this paper.

## Abbreviations

95%-CI or (. , .): 95%-confidence interval
μSv/h: Micro-Sievert per hour
Cs-134: Cesium-134
Cs-137: Cesium-137
Bq: Becquerel
FDNPP: Fukushima Daiichi Nuclear Power Plant
FHMS: Fukushima Health Management Survey
kBq/m^2^: Kilo-Becquerel per square meter
LBW: Low birth weight
LBWp: Low birth weight prevalence (or proportion)
MEXT: Ministry of Education, Culture, Sports, Science, and Technology in Japan
mSv: Milli-Sievert
OECD: Organization for Economic Co-operation and Development
OR: Odds Ratio
SAS: Statistical Analysis System, software produced by SAS Institute Inc.
TEPCO: Tokyo Electric Power Company
UNSCEAR: United Nations Scientific Committee on the Effects of Atomic Radiation
WHO: World Health Organization
